# Uptake and impact of facility‐based HIV self‐testing on PrEP delivery: a pilot study among young women in Kisumu, Kenya

**DOI:** 10.1002/jia2.25561

**Published:** 2020-08-20

**Authors:** Valentine Wanga, Victor Omollo, Elizabeth A Bukusi, Josephine B Odoyo, Jennifer F Morton, Lara Kidoguchi, Rachel Johnson, James P Hughes, Connie Celum, Jared M Baeten

**Affiliations:** ^1^ Department of Epidemiology University of Washington Seattle WA USA; ^2^ Department of Global Health University of Washington Seattle WA USA; ^3^ Centre for Microbiology Research Kenya Medical Research Institute Nairobi Kenya; ^4^ Departments of Obstetrics and Gynecology University of Washington Seattle WA USA; ^5^ Department of Biostatistics University of Washington Seattle WA USA; ^6^ Department of Medicine University of Washington Seattle WA USA

**Keywords:** PrEP, HIV testing, self‐testing, counselling, standard of care, satisfaction

## Abstract

**Introduction:**

HIV testing is a required part of delivery of pre‐exposure prophylaxis (PrEP) for HIV prevention. However, repeat testing can be challenging in busy, under‐staffed clinical settings, which could negatively impact PrEP uptake and continuation. We prospectively evaluated optional facility‐based HIV self‐testing (HIVST) among young women using PrEP in an implementation programme.

**Methods:**

Between February and November 2019, we collected data from young women receiving PrEP at two family planning facilities in Kisumu, Kenya. At each PrEP follow‐up visit, women were given the option to choose between provider‐initiated testing and HIVST. We assessed factors associated with HIVST uptake and compared satisfaction with HIV testing and clinic experience between acceptors and decliners of HIVST.

**Results:**

A total of 172 women were offered HIVST at 202 PrEP follow‐up visits. The median age was 21 years, 27% had multiple partners and 15% reported previously using HIVST. HIVST was accepted at 34.7% (70/202) of visits. Age (adjusted relative risk (aRR) 1.09 per year, 95% CI (confidence interval) 1.01 to 1.18), never being married (aRR 1.81, 95% CI 1.11 to 2.95) and having more PrEP follow‐up visits (aRR 1.13 per visit, 95% CI 1.04 to 1.23) were associated with HIVST uptake. Compared to HIVST decliners, HIVST acceptors were more likely to be very happy with their overall testing experience (73% vs. 47% of visits, *p* = 0.003) and were more likely to say they would use HIVST in the future (96% vs. 76%, *p* < 0.001). Women who accepted HIVST had shorter visits than those choosing standard provider‐initiated HIV testing (median [IQR]: 33 [32, 38] vs. 54 [41.5, 81] minutes, *p* = 0.003).

**Conclusions:**

In this pilot evaluation in Kenya, about one‐third of women using PrEP opted for HIVST over provider‐initiated testing, and those choosing HIVST spent less time in the clinic and were generally satisfied with their experience. HIVST in PrEP delivery is feasible and has the potential to simplify PrEP delivery and give clients testing autonomy. Additional studies are needed to explore optimal HIV retesting strategies in PrEP delivery, including the use of HIVST in PrEP at a larger scale and in different settings.

## 1 INTRODUCTION

As the scale‐up of pre‐exposure prophylaxis (PrEP) increases globally, barriers to PrEP implementation still remain. At the healthcare system level, a significant barrier is the complexity of PrEP delivery due to required regular HIV testing and monitoring in PrEP users [1, 2]. Integrating PrEP into routine primary care settings is feasible [3] and can facilitate reaching potential PrEP candidates, promote patient‐centred care, destigmatize PrEP and aid in dissemination of knowledge about PrEP to the broader community [4]. However, PrEP could add a burden on health systems, necessitating investigation of ways to simplify PrEP delivery.

Several studies in sub‐Saharan Africa have shown high acceptability, uptake and accurate use of HIV self‐testing (HIVST) in men and women in populations such as adolescents [5, 6, 7, 8], female sex workers and their partners [9, 10], and partners of women seeking antenatal and postnatal care [10, 11]. Compared to provider‐initiated testing and counselling, facility‐based HIVST has been shown to be highly acceptable among adolescents aged 15 to 24 years [8]. In the context of using PrEP, HIVST has been shown to be highly acceptable by mutually disclosed serodiscordant couples [12] and female sex workers who are interested in PrEP [13]. Nevertheless, most studies of HIVST have only evaluated HIVST for screening [7, 10, 12, 14, 15, 16, 17, 18] and to date, no study has evaluated the integration of facility‐based HIVST in PrEP delivery and its use as a tool to optimize patient flow and visit efficiency.

HIV testing is necessary before starting or restarting PrEP, and at least every 3 months during PrEP use; some guidelines additionally recommend testing 1 month after starting PrEP [19]. In busy and understaffed clinics, this volume of testing could create inefficiencies and negatively impact PrEP programmes, including reducing numbers willing to start or continue on PrEP due to long wait times and providers willing to prescribe PrEP. Therefore, incorporating strategies to streamline re‐testing may reduce staff time and associated costs [20] and improve PrEP delivery efficiency. We conducted an evaluation of facility‐based HIVST aimed to streamline PrEP delivery. We measured HIVST uptake, assessed factors associated with HIVST uptake, evaluated satisfaction with PrEP delivery and the impact of HIVST on duration of PrEP delivery processes.

## 2 METHODS

### 2.1 Study setting, population and design

Prevention Options for Women Evaluation Research (POWER) is an ongoing implementation science study evaluating PrEP delivery to young women in Kenya and South Africa [21]. We enrolled women attending PrEP follow‐up visits (scheduled for every 3 months) at the two POWER study sites – both family planning clinics – in Kisumu, Kenya (Jaramogi Oginga Odinga Teaching and Referral Hospital and Kisumu Medical Education Trust). HIVST is broadly supported by the Kenya Ministry of Health and thus this pilot was designed for evaluation of HIVST at the Kenya POWER clinics. Eligibility criteria for POWER cohort were age 16 to 25 years, able and willing to provide written informed consent, recently sexually active (having had vaginal intercourse at least once in the previous 3 months) and HIV uninfected based on a negative HIV rapid test on the date of enrolment.

We evaluated our study outcomes in two periods: a “standard of care period” (14 weeks), during which outcomes were evaluated under standard of care with no HIVST offered, and a subsequent “HIVST period” (17 weeks), during which women were given the option to choose between provider‐initiated testing and counselling (PITC) and HIVST. We used the OraQuick® HIV Self‐Test (OraSure Technologies, USA) kit, one of the three test kits that have been approved for HIVST by the Kenya Ministry of Health [22]. OraQuick® detects HIV 1/2 antibodies in oral fluid (mouth swab/saliva) samples and has been shown to have high sensitivity (87.9%) and specificity (98.0%) when used by lay individuals in Kenya [23].

### 2.2 Ethical considerations

The POWER study protocol was reviewed and approved by the Institutional Review Boards (IRBs) at the University of Washington and the Human Subjects Review Committees at each clinical site. We obtained approval for the protocol for the HIVST study and other related documents from the IRBs at the University of Washington and the Kenya Medical Research Institute. The study included women of ages 16 to 25 years (an age‐group that contributes a large proportion of the burden of HIV in Sub‐Saharan Africa), and we followed local guidelines for consent for those under 18 years of age.

### 2.3 Data collection and outcomes

#### 2.3.1 Questionnaires

We collected data between February and November 2019. At the end of each visit, participants completed a standardized questionnaire, administered by a researcher not involved in care provision for the participants, to assess demographics and study outcomes. Questionnaires were administered only to women who consented to be enrolled in the HIVST pilot, agreed to take the questionnaire and had not tested for HIV elsewhere in the clinic on the same day during the HIVST period. Individual‐level data on why women did not enrol in the HIVST pilot were not collected.

#### 2.3.2 Time‐and‐motion

We conducted time‐and‐motion observations during two‐week periods in March, May and October 2019. After the study nurse asked the participant for permission to be observed, a research assistant not involved in care provision observed participants in the order in which they attended the clinic, from their arrival to exit from the clinic; those who arrived during an ongoing observation were not observed due to limited personnel capacity. Time for questionnaires completion was not observed as these would not occur in real‐world PrEP delivery. Additionally, for privacy and comfort of participants, time for confirmatory testing was not observed.

#### 2.3.3 HIVST and results

Those who chose HIVST were each given a timer and self‐test kit and directed to a private location near the POWER study room where detailed HIVST instructions were posted on the wall in three languages (English, Kiswahili and Luo). We used an unassisted HIVST approach, but participants could ask providers for clarifications if needed. The participants left the self‐test to run in lockable cabinets while continuing with other PrEP delivery procedures. At completion of HIVST, participants brought the kits to a provider and verbally reported their results. The provider recorded the participant’s results and his/her interpretation of the results. If a HIVST result was positive, participants were tested per the POWER study protocol and requested to return to the clinic after one month for follow‐up testing.

#### 2.3.4 Outcomes

The primary outcomes of interest were HIVST uptake, testing experience (how easy testing was, how easy it was to understand test results, what was liked most about testing and how long waited for testing), satisfaction with testing (how happy with overall HIV testing and whether would recommend HIVST in the future), satisfaction with clinic visit (how rated overall clinic experience and how happy with: how treated in clinic, clarity of explanations given by providers, time given to ask questions, involvement in making decisions about PrEP use, clinic waiting time) and duration of key PrEP delivery procedures (HIV testing, counselling, PrEP dispensing and waiting). We also assessed reasons for testing choice (PITC or HIVST), and for considering HIVST in the future.

#### 2.3.5 Statistical analyses

We described the number and proportion of women who accepted HIVST, and response categories for each experience and satisfaction outcome by study period. We used log‐Poisson generalized estimating equations (GEE) with robust standard errors and independence correlation structure to assess factors related to HIVST uptake. We present relative risks (RRs) adjusted for site and baseline covariates identified *a priori* (age, education, marital status, multiple partnerships, baseline prior use of HIVST and number of follow‐up visits in the POWER cohort at enrolment in this study) [17, 24]. To compare differences in experience and satisfaction between acceptors and decliners of HIVST, we performed score tests of exposure (questionnaire item) coefficients from site‐adjusted log‐Poisson GEE models with robust standard errors and independence correlation structure. To assess the impact of HIVST on duration of PrEP procedures, we described the median and interquartile range of the duration of each PrEP delivery procedure and used the Wilcox test to compare times between acceptors and decliners of HIVST; as a sensitivity analysis, we repeated the comparison excluding outlying waiting times longer than 30 minutes. We used two‐sided *p*‐values and considered them significant if <0.05. We used SAS version 9.4 (SAS Institute Inc., Cary, NC, USA) and R version 3.6.1 (www.r-project.org) for analyses.

## 3 RESULTS

### 3.1 Participant characteristics

Overall, 249 women contributed 362 PrEP follow‐up visits in this study: 160 visits (148 women) during the standard of care period and 202 visits (172 women) in the HIVST period, with a maximum of three observations per person. Seventy‐one (28.5%) women had at least one visit during both periods, 12 had two visits during the standard of care period and 30 had two visits during the HIVST period. In the HIVST study data collection period, 279 women contributed 441 follow‐up visits in the main POWER study; reasons why women did not enrol in the HIVST study were principally lack of time or having already been tested at the routine HIV Testing Services point prior to arrival for POWER follow‐up. Among those enrolled in the HIVST pilot, the median age was 21 years (interquartile range [IQR]) [19, 23], most women had never married (69.1%), had only one partner (72.6%) and had completed up to secondary school (41.8%) (Table 1). At enrolment in this study, most women (85.5%) also reported that they had never used HIVST (Table 1).

**Table 1:** Characteristics of women (overall and by HIVST period), and predictors of HIVST uptake

Variable	Overall^a^ (N = 249)	HIV self‐testing acceptors^a^ (N = 55)	HIV self‐testing decliners^a^ (N = 117)	Predictors of HIV self‐testing uptake during HIV self‐testing period (N^b^ = 202)	
				Adjusted RR^c^ (95% CI)	*p*‐value
Age (years)	21.0 [19.0, 23.0]	22.0 [19.0, 23.5]	21.0 [19.0, 23.0]	1.09 (1.01, 1.18)	0.025
Highest education completed					
Primary	81 (32.5)	18 (32.7)	42 (35.9)	1.12 (0.49, 2.58)	0.718
Secondary	104 (41.8)	19 (34.5)	47 (40.2)	0.99 (0.42, 2.31)	0.978
Tertiary	25 (10.0)	8 (14.5)	12 (10.3)	1.29 (0.52, 3.21)	0.579
Vocational training	22 (8.8)	6 (10.9)	7 (6.0)	1.33 (0.55, 3.22)	0.522
No schooling completed	17 (6.8)	4 (7.3)	9 (7.7)	Reference	
Marital status					
Never married	172 (69.1)	40 (72.7)	72 (61.5)	1.81 (1.11, 2.95)	0.017
Ever married	77 (30.9)	15 (27.3)	45 (38.5)	Reference	
Number of current sex partners					
Only 1 partner	180 (72.6)	37 (67.3)	82 (70.7)	1.02 (0.69, 1.53)	0.894
>1 partner	68 (27.4)	18 (32.7)	34 (29.3)	Reference	
Used HIVST in the past					
Yes	36 (14.5)	8 (14.5)	19 (16.2)	0.94 (0.61, 1.46)	0.781
No	213 (85.5)	47 (85.5)	98 (83.8)	Reference	
Number of POWER cohort follow‐up visits at enrolment in the HIVST study	1.0 [0.0, 3.0]	2.0 [0.0, 4.0]	1.0 [0.0, 3.0]	1.13 (1.04, 1.23)	0.005

HIVST, HIV self‐testing.

^a^ Number (N) (%) or median [interquartile range (IQR)]

^b^ Number of observations by acceptors (70) and decliners (132)

^c^ Adjusted for site and other variables (age, marital status, education, baseline prior use of HIVST and number of POWER cohort follow‐up visits at enrolment in the HIVST study).

### 3.2 Uptake of HIVST

During the HIVST period, HIVST was accepted at 70 (34.7%) of 202 visits (Table 2). Of the 172 women who had a visit during the HIVST period, 55 (32.0%) accepted HIVST at their first opportunity; among 30 who attended clinic twice during the HIVST period, six accepted and 12 declined HIVST at two consecutive visits, three initially accepted then later declined HIVST and nine declined then later accepted HIVST.

**Table 2:** HIV testing experience and satisfaction

Item	Standard of care period (n = 160), N (%)	HIV self‐testing period		
		Acceptors (n = 70), N (%)	Decliners (n = 132), N (%)	*p*‐value^a^
Ever self‐tested for HIV in the past				
Yes	22 (13.8)	14 (20.0)	25 (18.9)	0.871
No	138 (86.2)	56 (80.0)	107 (81.1)	
If ever self‐tested, where participant got a self‐test kit				
Research study	13 (59.1)	8 (57.1)	13 (52.0)	0.943
Facility	6 (27.3)	3 (21.4)	5 (20.0)	
Pharmacy/friend/family	3 (13.6)	3 (21.4)	7 (28.0)	
Whether participant would consider (or repeat) HIV self‐testing in the future				
Yes	138 (86.2)	67 (95.7)	100 (75.8)	<0.001
No	22 (13.8)	3 (4.3)	32 (24.2)	
Main reason to consider HIV self‐testing in the future				
Privacy/confidentiality	30 (21.7)	NA	26 (26.0)	NA
Personal empowerment/taking charge of my own health	72 (52.2)	NA	51 (51.0)	
No pricking/painless	16 (11.6)	NA	8 (8.0)	
Saves time/no waiting in queues	18 (13.0)	NA	11 (11.0)	
Other	2 (1.4)	NA	4 (4.0)	
If self‐tested, main reason to not want to self‐test again				
Testing difficult to do	NA	0 (0.0)	NA	NA
I made mistakes when doing the test	NA	0 (0.0)	NA	
I did not understand the results	NA	1 (33.3)	NA	
I don’t believe the results/still have to go for confirmatory testing	NA	0 (0.0)	NA	
Afraid/prefer to have counsellor with me	NA	2 (66.7)	NA	
Main reason why declined HIV self‐testing				
I was afraid the test would be difficult to do	NA	NA	22 (16.7)	NA
I was not comfortable testing alone	NA	NA	44 (33.3)	
The HTS queue was not long	NA	NA	2 (1.5)	
I did not trust self‐testing	NA	NA	18 (13.6)	
I did not know how to use HIV self‐testing kit	NA	NA	15 (11.4)	
I am used to provider‐initiated testing and counselling	NA	NA	13 (9.8)	
Provider‐initiated testing and counselling is faster	NA	NA	9 (6.8)	
Other	NA	NA	9 (6.8)	
How easy participant found HIV testing experience/conducting of HIV self‐testing^b^				
Very difficult	0 (0.0)	1 (1.4)	0 (0.0)	0.061
Difficult	6 (3.8)	3 (4.3)	2 (1.5)	
Undecided	2 (1.2)	2 (2.9)	1 (0.8)	
Easy	97 (60.6)	36 (51.4)	92 (69.7)	
Very easy	55 (34.4)	28 (40.0)	37 (28.0)	
How easy it was to understand test results^b^				
Very difficult	0 (0.0)	0 (0.0)	0 (0.0)	0.129
Difficult	7 (4.4)	6 (8.7)	1 (0.8)	
Undecided	5 (3.1)	1 (1.4)	3 (2.3)	
Easy	93 (58.1)	37 (53.6)	87 (65.9)	
Very easy	55 (34.4)	25 (36.2)	41 (31.1)	
What was liked most about provider‐initiated HIV testing and counselling				
Having someone with me during the test	34 (21.2)	NA	23 (17.4)	NA
Getting counselling during the testing	73 (45.6)	NA	83 (62.9)	
Getting help understanding my testresults	45 (28.1)	NA	17 (12.9)	
Other	8 (5.0)	NA	9 (6.8)	
What was liked most about HIV self‐testing				
Privacy/confidentiality	NA	24 (34.3)	NA	NA
Personal empowerment/taking charge of my own health	NA	17 (24.3)	NA	
No pricking/painless	NA	17 (24.3)	NA	
Saves time/no waiting in queues	NA	8 (11.4)	NA	
Other	NA	4 (5.7)	NA	
How long participant waited for HIV testing or HIV self‐testing kit				
0 to 15 minutes	133 (83.1)	54 (77.1)	118 (89.4)	0.081
16 to 30 minutes	17 (10.6)	16 (22.9)	14 (10.6)	
>30 minutes	10 (6.2)	0 (0.0)	0 (0.0)	
Would you recommend HIV self‐testing				
Yes	NA	67 (95.7)	NA	NA
No	NA	3 (4.3)	NA	
How happy participant was with overall testing experience^c^				
Very unhappy	0 (0.0)	1 (1.4)	0 (0.0)	0.003
Unhappy	1 (0.6)	1 (1.4)	1 (0.8)	
Undecided	1 (0.6)	1 (1.4)	2 (1.5)	
Happy	44 (27.5)	16 (22.9)	66 (50.0)	
Very happy	114 (71.2)	51 (72.9)	63 (47.4)	

HTS, HIV testing services; HIVST, HIV self‐testing; PITC, provider‐initiated testing and counselling.

^a^ p‐value comparing acceptors and decliners

^b^ For HIVST period comparisons, collapsed into three categories: very difficult/difficult/undecided, easy and very easy

^c^ For HIVST period comparisons, collapsed into three categories: very unhappy/unhappy/undecided, happy and very happy

Older age (adjusted risk ratio (aRR) 1.09 per year, 95% CI (confidence interval) 1.01 to 1.18, *p* = 0.025), being never married (aRR 1.81, 95% CI 1.11 to 2.95, *p* = 0.017) and having more PrEP follow‐up visits (aRR 1.13 per visit, 95% CI 1.04 to 1.23, *p* = 0.005) were associated with an increased chance of HIVST uptake (Table 1). Highest education completed, multiple partnerships and prior use of HIVST were not significantly associated with uptake of HIVST.

Of the 70 HIV self‐tests conducted, 68 were successfully completed (in one case, the participant’s child spilled the reagents and HIVST was abandoned, in the other case the participant chose to not complete HIVST and reverted to PITC). Of the 68 tests, four (5.9%) were positive, whereas one (1.5%) was invalid/indeterminate. Follow‐up testing of these five cases by a trained provider on the same day, per the Kenyan national HIV testing algorithm, yielded negative results. Based on the provider’s reading of HIVST results, 64 (94%) of the successfully completed tests were correctly read and interpreted by participants. In one test, a HIVST result was positive, but the participant reported an indeterminate result. In two tests, HIVST results were positive, but the participants reported that they did not know their results. Lastly, in one test, a HIVST result was negative, but a participant reported that she did not know her results.

### 3.3 Testing experience and satisfaction

In general, women were either happy or very happy (98% visits) with their overall testing experience (Table 2). Most women found their testing easy/very easy (95% visits) and their HIV test results easy/very easy to understand (94% visits). For HIVST acceptors, privacy/confidentiality was what they cited liking most about HIVST (34%), and in 96% of acceptor visits, women said they would repeat HIVST in the future and recommend HIVST to others. For HIVST decliners, not being comfortable with testing alone was the main reason for declining HIVST (33% visits), getting counselling during testing was liked most about PITC (63% visits), and personal empowerment/taking charge of ones’ health was the main reason to consider HIVST in the future (51%). Surprisingly, three of the women who had a false‐positive HIVST result said they would repeat HIVST in the future.

Comparing HIVST acceptors and HIVST decliners, there was no statistically significant difference in testing experience, prior use of HIVST, ease of understanding test results and time spent waiting for HIV testing/HIVST kit. Still, HIVST acceptors found their HIV testing experience very easy compared to HIVST decliners (40% visits vs. 28% visits, *p* = 0.061). HIVST acceptors were more likely to be very happy with their overall testing experience than decliners (73% visits vs. 47% visits, *p* = 0.003). Finally, HIVST acceptors were more likely to say they would repeat HIVST than decliners were to say they would consider HIVST in the future (96% visits vs. 76% visits, *p* < 0.001).

### 3.4 Clinic experience and satisfaction

There were no statistically significant differences in satisfaction with the clinic visit experience between HIVST acceptors and HIVST decliners (*p*> 0.05) (Table 3). In most visits, women were happy/very happy (>90% of visits in each period) with their clinic experience, including how they were treated, the clarity of explanations given by providers, the time they had to ask questions, their involvement in making decisions about PrEP use, and how long they had to wait in the clinic. Likewise, in most visits, women rated their overall clinic experience as good (96% visits) (Table 3).

**Table 3:** Clinic experience and satisfaction

Item	Standard of care (n = 160), N (%)	HIV self‐testing period		
		Acceptors (n = 70), N (%)	Decliners (n = 132), N (%)	*p*‐value^a^
How happy participant was with the way she was treated at the clinic^b^				
Very unhappy	0 (0.0)	1 (1.4)	4 (3.0)	0.187
Unhappy	1 (0.6)	0 (0.0)	0 (0.0)	
Undecided	0 (0.0)	0 (0.0)	0 (0.0)	
Happy	47 (29.4)	19 (27.1)	51 (38.6)	
Very happy	112 (70.0)	50 (71.4)	77 (58.3)	
How happy participant was with the clarity of all explanations given by providers^b^				
Very unhappy	0 (0.0)	1 (1.4)	0 (0.0)	0.276
Unhappy	1 (0.6)	1 (1.4)	0 (0.0)	
Undecided	1 (0.6)	0 (0.0)	1 (0.8)	
Happy	53 (33.1)	24 (34.3)	60 (45.5)	
Very happy	105 (65.6)	44 (62.9)	71 (53.8)	
How happy participant was with the time she had to ask providers questions^b^				
Very unhappy	0 (0.0)	1 (1.4)	2 (1.5)	0.965
Unhappy	3 (1.9)	1 (1.4)	0 (0.0)	
Undecided	4 (2.5)	1 (1.4)	2 (1.5)	
Happy	80 (50.0)	35 (50.0)	68 (51.5)	
Very happy	73 (45.6)	32 (45.7)	60 (45.5)	
How happy participant was with her involvement in making decisions about PrEP use^b^				
Very unhappy	0 (0.0)	1 (1.4)	0 (0.0)	0.491
Unhappy	0 (0.0)	1 (1.4)	1 (0.8)	
Undecided	6 (3.8)	1 (1.4)	5 (3.8)	
Happy	55 (34.4)	22 (31.4)	54 (40.9)	
Very happy	99 (61.9)	45 (64.3)	72 (54.5)	
How happy participant was with how long she had to wait in the clinic^b^				
Very unhappy	3 (1.9)	1 (1.4)	0 (0.0)	0.079
Unhappy	9 (5.6)	3 (4.3)	6 (4.5)	
Undecided	2 (1.2)	0 (0.0)	2 (1.5)	
Happy	73 (45.6)	25 (35.7)	68 (51.5)	
Very happy	73 (45.6)	41 (58.6)	56 (42.4)	
How participant would rate her overall experience at the clinic^c^				
Poor	0 (0.0)	0 (0.0)	0 (0.0)	0.637
Fair	2 (2.9)	6 (4.5)	6 (4.5)	
Good	28 (40.0)	60 (45.1)	59 (44.7)	
Excellent	40 (57.1)	67 (50.4)	67 (50.8)	

HIVST, HIV self‐testing; PrEP, pre‐exposure prophylaxis.

^a^ *p*‐value comparing acceptors and decliners

^b^ For HIVST period comparisons, collapsed into three categories: very unhappy/unhappy/undecided, happy and very happy

^c^ For HIVST period comparisons, collapsed into three categories: poor/fair, good and excellent

### 3.5 HIVST and PrEP delivery processes

We observed 40 participants (10 per clinical site per period) – during the HIVST period, we observed nine HIVST acceptors and 11 HIVST decliners. Total visit time was shorter for those using HIVST compared to those who declined HIVST (median 33 vs. 54 minutes, *p* = 0.003) (Table 4), and total visit time during standard of care period was comparable to that of HIVST decliners (median 55 vs. 54 minutes). HIV testing itself was longer for HIVST than PITC (median [IQR]: 27 [26, 30] vs. 15 [13, 17.5] minutes, *p* = 0.001) and PrEP dispensing time was slightly shorter for HIVST acceptors than decliners (median [IQR]: 2.5 [1, 3] vs. 4 [3.3, 4], *p* = 0.01). Median waiting time was longer for HIVST decliners than HIVST acceptors (11 vs. 2 minutes), although this difference was not statistically significant. In sensitivity analysis omitting waiting times longer than 30 minutes, the total time spent in the clinic was still shorter for HIVST acceptors than HIVST decliners (median 33 vs. 46 minutes, *p* = 0.014).

**Table 4:** Duration (minutes) of PrEP delivery procedures

	HIV self‐testing acceptors^a^	HIV self‐testing decliners (tested by a provider)^a^	*p*‐value
HIV testing	27.0 [26.0, 30.0]	15.0 [13.0, 17.5]	0.001
Counselling^b^	8.5 [5.0, 10.0]	8.0 [5.3, 9.5]	0.892
PrEP dispensing	2.5 [1.0, 3.0]	4.0 [3.3, 4.0]	0.010
Other^c^	7.0 [5.0, 9.0]	6.0 [4.5, 12.5]	0.646
Client waiting time	2.0 [1.0, 6.0]	11.0 [0.5, 25.0]	0.301
Total visit time^d^	33.0 [32.0, 38.0]	54.0 [41.5, 81.0]	0.003

Represent time from the beginning to the end of the specific procedure. PrEP, pre‐exposure prophylaxis.

^a^ Median [IQR]

^b^ Counselling on PrEP (adherence, side effects), risk reduction, family planning, sexually transmitted infections

^c^ Other procedures include confirmation of client ID, taking vitals (blood pressure, pulse, height and weight) and scheduling next appointment date

^d^ Total visit time includes time from entry to exit from the clinic while visit times for procedures.

## 4 DISCUSSION

In this pilot study of HIVST in PrEP delivery, women chose HIVST over PITC at about one‐third of visits, successfully completed HIVST in 97% of visits, and correctly read their HIVST results 94% of the time. In general, women were satisfied with their HIV testing and clinic experience, and compared to HIVST decliners, HIVST acceptors were more likely to say they would use HIVST in the future. Age, marital status and number of PrEP visits were associated with uptake of HIVST, and HIVST decliners spent more time at the clinic than HIVST acceptors. Consistent with other studies, we found that personal empowerment/taking charge of ones’ health was the main reason for considering HIVST in the future [12, 25], and that privacy/confidentiality was what was liked most about HIVST [26].

Four women incorrectly interpreted their results in this study; three had false‐positive HIVST results, with one reporting an indeterminate result and two reporting not knowing their results, whereas one had a negative HIVST result, but reported not knowing her result. The anxiety of getting a positive result possibly influenced the women’s ability to correctly interpret their HIVST results, highlighting an advantage of facility‐based HIVST in PrEP delivery, where a provider can verify test results, provide counselling, perform confirmatory testing and closely follow‐up with a client who has a positive HIVST result, as was done in this study. More importantly, considerations about the emotional consequences of false‐positive results and incorrect interpretation of HIVST results warrant additional evaluation of the use of HIVST in PrEP delivery.

The current World Health Organization guidelines do not recommend the use of HIVST by people taking PrEP [27] due to concerns about false‐negative results. Taking PrEP with undetected HIV could lead to drug resistance, but a recent review of PrEP studies found that resistance selection for tenofovir disoproxil fumarate and emtricitabine with PrEP use is infrequent [28]. Evaluations of HIV rapid diagnostic tests (RDTs) among PrEP users have found variable sensitivities and specificities, with specificities >98% [29, 30, 31, 32, 33]. An evaluation of OraQuick ADVANCE^®^ Rapid HIV‐1/2, the kit we used in our study, found that it had high specificity (99.99%) and high negative predictive value (99.94%) [32]. Still, considerations about the performance of RDTs and drug resistance with undiagnosed HIV infection necessitate additional evidence on the safety and utility of HIVST in PrEP delivery.

Integrating PrEP delivery within family planning clinics could promote PrEP screening and uptake, but it could also add delivery challenges to providers and clients if not combined with additional capacity and resources. For providers, adding PrEP increases the number of people to be tested for HIV and screening and counselling time, which could lower the quality of care provided. In a qualitative study among HIV care providers in Western Kenya, high strain due to high patient volume was reported as a barrier to providing high‐quality patient care [34]. For clients, long waiting times in clinics could discourage PrEP continuation and utilization of other services; long waiting times have been cited as a barrier to using HIV services among people living with HIV [35, 36], and for PrEP clients who are not sick to begin with, this could further dissuade care‐seeking. Our finding that HIVST acceptors spent less time at the clinic than HIVST decliners suggests that HIVST could shorten PrEP refill visits and promote PrEP continuation.

Unlike the sequential pattern of PrEP procedures for those who chose PITC, HIVST acceptors continued with other PrEP procedures (taking vitals and counselling) while waiting for HIVST to run; this contributed to the observed difference in total time spent in clinic. With multiple use of HIVST, we believe that women would become more familiar with HIVST procedures, and the observed HIVST median time of 27 minutes would be reduced. PrEP users in sub‐Saharan Africa have reported that practicalities of PrEP use such as finding time for appointments and service delivery environment (including clinical staff capacity) play a key role in their considerations for PrEP use [37]. HIVST reduced the overall time spent in clinic in our study, further underscoring the potential for HIVST to streamline PrEP visits in busy and understaffed clinics and promote PrEP persistence.

The average age in this study was 21 years. Adolescents girls and young women (AGYW, aged 15 to 24 years) in sub‐Saharan Africa have expressed the need for HIV prevention options that consider different facets of their lives such as their risk perception, relationships dynamics, concerns about PrEP side effects and burden of PrEP use [37]. We expected at least a 50% uptake of HIVST based on results from previous HIVST studies among AGYW [5, 6, 7, 8], but found that a third of women preferred HIVST, whereas the rest preferred PITC. A study among adolescents also found that over 80% preferred directly assisted facility‐based HIVST over PITC [7] – it is possible that directly assisted HIVST for first‐time users could have improved HIVST uptake in our study. Our finding that having more PrEP follow‐up visits pre‐enrolment in the HIVST study was associated with choosing HIVST suggests that women who had repeatedly used PITC knew what to expect during HIV testing and were comfortable testing alone during subsequent PrEP visits. Thus, HIVST could be used as an approach to expand retesting options for women in PrEP programmes.

This was a pilot evaluation and it has important limitations. The main limitation of this study is the short duration of the HIVST period; those who initially declined HIVST might have chosen HIVST had they been given more opportunity to do so. Similarly, we were unable to more fully assess reasons for accepting or declining HIVST, and vice versa, over time. We introduced unassisted HIVST among women who were already enrolled in a PrEP programme and accustomed to PITC; a programme that used HIVST from the start might have found greater uptake. Third, we used "happiness" to measure satisfaction with overall HIV testing and clinic experience. Although “happiness” and “satisfaction” both imply contentment to some extent, standardized instruments that measure satisfaction [38] might have provided more precise information and would be important to utilize for future studies of HIVST implementation. Finally, women participating in this evaluation were enrolled in a research study and HIVST in more programmatic settings could find different results; importantly, procedures in our study such as PITC, laboratory testing and PrEP dispensing were integrated into standard clinic procedures, allowing for their evaluation within existing clinical settings. Despite these potential limitations, our study provides important evidence on the feasibility, uptake and impact of HIVST in PrEP delivery, and is the first study to investigate unassisted facility‐based HIVST in a PrEP context.

## 5 CONCLUSIONS

This pilot evaluation of HIVST in the context of PrEP shows that facility‐based HIVST in PrEP delivery is feasible and can reduce visit times. More studies are needed to understand how HIVST could be used not only to screen for HIV but also as a tool to streamline PrEP delivery, promote PrEP persistence and offer the flexibility for PrEP users to have testing options during their PrEP visits.

## COMPETING INTEREST

All authors have no conflict of interest related to this work.

## AUTHORS’ CONTRIBUTIONS

VW and JMB contributed to study conception and design. VW, VO, LK and JFM contributed to data collection, data management and study coordination. VW, JPH and JMB contributed to analysis and interpretation of the data. VW and JMB contributed to drafting of the manuscript. VW, VO, EAB, JBO, JFM, LK, RJ, JPH, CC and JMB contributed to critical revision of the manuscript for important intellectual content. All authors reviewed and approved the final version of the manuscript.
